# Common gene mutations in 103 authenticated colorectal cancer cell lines

**DOI:** 10.1038/s41389-026-00599-0

**Published:** 2026-01-27

**Authors:** Christian Kranjec, Ina A. Eilertsen, Luís Nunes, Seyed H. Moosavi, Kaja C. G. Berg, Mette Eknæs, Merete Hektoen, Barbara Niederdorfer, Guro E. Lind, Rolf I. Skotheim, Anita Sveen, Ragnhild A. Lothe

**Affiliations:** 1 Department of Molecular Oncology, Institute for Cancer Research, Oslo University Hospital, Oslo, Norway; 2 Department of Biosciences, The Faculty of Mathematics and Natural Sciences, University of Oslo, Oslo, Norway; 3 Department of Informatics, The Faculty of Mathematics and Natural Sciences, University of Oslo, Oslo, Norway; 4 Institute of Clinical Medicine, Faculty of Medicine, University of Oslo, Oslo, Norway

**Keywords:** Cancer models, Genomic instability, Cancer genetics, Gene amplification

## Abstract

Colorectal cancer (CRC) cell lines represent the main molecular subtypes of tumors and are valuable models for preclinical investigations. However, cell lines can diverge over time and careful selection of models based on their molecular features is key. We have authenticated 103 commonly used CRC cell lines and present the mutation profiles of 20 CRC-relevant genes sequenced to an average depth of 575 times coverage. The cell lines reflected the distinct mutation patterns of hypermutation phenotypes associated with microsatellite instability and pathogenic *POLE* mutations. Hypermutated cell lines appeared to have a stronger mutational divergence and more frequent subclonal mutations, while mutations not associated with hypermutation were more frequently homozygous or hemizygous, classified as pathogenic, and subject to stronger selection pressure. Loss of heterozygosity at mutated loci was primarily observed in tumor suppressor genes. Genetic interactions based on co-occurring mutations identified cell lines representative of particularly aggressive subtypes of CRC, including concurrent *BRAF* p.V600 and truncating *APC* mutations, as well as *APC*/*TP53*/*RAS* triple mutations with double hits of *APC*. This study provides a resource to guide the selection of cell lines for functional studies of CRC, and detailed mutation data including classifications of pathogenicity, variant allele frequencies and illustrations of the mutation distribution along the length of encoded proteins are included.

## Introduction

Cell lines are widely used as preclinical models in cancer research. Cell cultures are amenable to experimental perturbations and provide an indefinite source of biological material. Cell lines derived from human tumors and cultured following stringent criteria can retain several characteristics of the cancer of origin [1–3] and have been used for biomarker discovery and to model genotype/phenotype associations with drug sensitivity [4–10]. However, cell lines have important limitations as models of complex diseases such as solid cancers. The cell cultures are commonly grown in two-dimensional monolayers and fail to retain the native tissue architecture, as well as interactions with the extracellular matrix and diverse cell types of the tumor microenvironment. In addition, most solid cancers have a high degree of tumor heterogeneity, and cell lines of monoclonal origin fail to capture this diversity. Many conventional cell lines have been cultured over several years and are also likely to have diverged from the original tumor because of adaptation to culturing conditions, evolution, and genetic drift. It is therefore important to carefully select suitable cell line models for each specific research question. The fidelity of cell lines for various traits of the original tumor is highly variable among cancer types [11, 12]. Cell lines of colorectal cancers (CRCs) compare favorably with many other cancer types in this respect [13].

Mutation profiles of CRCs are shaped by different types of genomic instability. Approximately 80% of CRCs have chromosomal instability and frequent DNA copy number variations (CNVs). Nearly all these tumors have activation of the WNT signaling pathway due to inactivating mutations of *APC* or, less frequently, by activating mutations of *CTNNB1*. However, the rate of single-nucleotide variants (SNVs) is moderate compared to other solid cancer types, and only a small subset of the mutations is currently clinically “actionable” [14, 15]. Mutation-guided treatment options include anti-EGFR antibodies in *KRAS*/*NRAS* (*RAS*) wild-type tumors and drug combinations targeting *BRAF* p.V600E mutations [8, 9, 16] or *ERBB2* amplification and overexpression [17, 18]. Co-occurring or mutually exclusive mutations can further pinpoint clinically relevant subgroups, as illustrated with *RAS* and *TP53* co-mutations in an aggressive subgroup of metastatic CRCs [19, 20]. Other subtypes of CRCs are characterized by hypermutation phenotypes, either microsatellite instability (MSI) and frequent insertions-deletions (indels) caused by DNA mismatch repair deficiency [21], or frequent SNVs caused by pathogenic mutations in the proofreading domain of *POLE* [22]. Both hypermutation phenotypes are immunogenic and vulnerable to immune checkpoint inhibitors [23].

In vitro investigations have contributed significantly to the development of mutation-guided treatment options for patients with CRC [24, 25]. To expand the resource of information on genomic profiles beyond previous studies [26–32], we report the mutation profiles of 20 CRC-relevant genes in 103 authenticated CRC cell lines.

## Results

### Genomic phenotypes of the CRC cell line collection

The cell line collection included commonly used in vitro models of CRC (*n* = 103; Supplementary Table 1). All were derived from colorectal adenocarcinomas, except three with neuroendocrine tumor origin and distinct morphological and molecular features (COLO 320, HROC57, and NCI-H716) [33–36]. The majority derived from primary tumors (78%; *n* = 64 colonic and 16 rectal tumors) and the rest from diverse metastatic sites (20%). Two patient-matched primary-metastasis cell line pairs were included, Isreco-1/Isreco-3 and SW480/SW620. Short tandem repeat (STR) profiling further confirmed that DLD-1 is derived from HCT 15 and WiDr from HT-29. All other cell lines had unique STR profiles with a median match of 100% (range 77%-100%) to the correct reference according to the Cellosaurus STR Similarity Search (CLASTR) tool. HROC112Met T0 M2 has been discontinued by the supplier, and no updated STR reference profile was available. All STR profiles across 16 markers are included in Supplementary Table 2. The cell lines were analyzed after a median of 5 passages (range 3–9; Supplementary Table S1).

**Fig. 1:**
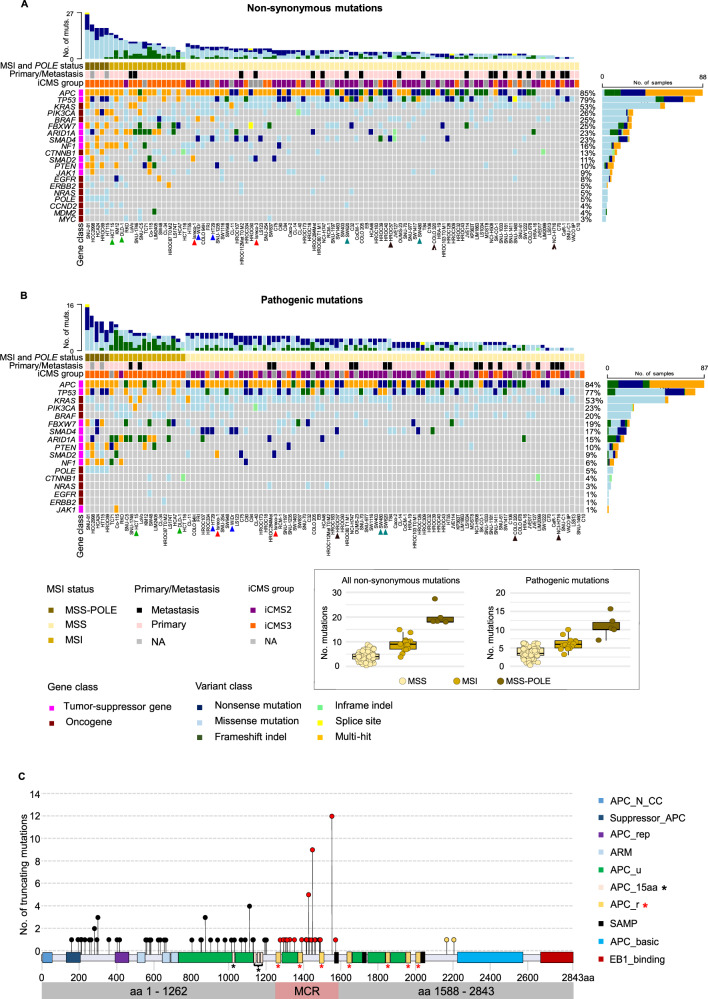
Landscape of SNVs and indels of CRC cell lines. **A** Oncoplot of non-synonymous SNVs and indels of 20 CRC-critical genes in 103 CRC cell lines (details in Supplementary Table 9 and 10). Genes (rows) and cell lines (columns) are ordered based on mutation frequency and MSI/*POLE* status of the cell lines. Mutations are color-coded according to variant class. Multi-hit mutations (orange) indicate multiple mutations of the same gene in the same cell line. The bar plots on the right side and top represent the number of mutated samples per gene and mutated genes per sample, respectively. The sample annotation bars indicate the MSI/*POLE* status and primary or metastatic tumor origin, and the gene class annotation indicates tumor-suppressors and oncogenes. Cell lines derived from the same patient (DLD-1:HCT 15, Isreco-1:Isreco-3, HT-29:WiDr, SW480:SW620) are marked by arrowheads of the same color. Cell lines derived from neuroendocrine tumors (COLO 320, HROC57, NCI-H716) are marked with black arrowheads. **B** Same plot as in (**A**), including only mutations classified as pathogenic or likely pathogenic in Supplementary Table 9. MSI/MSS: microsatellite instable/stable; MSS-POLE: *POLE* mutated samples. **C** Lollipop plot of truncating multi-hit mutations of *APC* across 42 cell lines (Supplementary Table 12). Mutations (lollipop circles) are colored according to whether they occur upstream (aa 1-1262 - black), within (MCR, aa 1263-1587 - red), or downstream (aa 1588-2843 - yellow) the MCR. Protein domains are color-coded and described according to Pfam nomenclature (Supplementary Table 11). Black and red asterisks mark the position of 15- and 20-amino acid β-catenin binding sites, respectively.

A 20-gene panel was selected based on mutation frequency and relevance in CRC (Methods) and sequenced to a median depth of 575x per cell line (10–90th) percentile range 457x-683x; Supplementary Table 3). Non-synonymous SNVs and indels were detected in all cell lines except C10 (Fig. 1A). Previous studies have reported a missense mutation of *TP53* (c.733 G > A) in C10 [37], but we and others [27] found no such evidence.

The cell lines reflected the distribution of the MSI (*n* = 16, 16%) and microsatellite stable (MSS; *n* = 87, 84%) phenotypes of sporadic CRC (Supplementary Table 1). MSI was more frequent among cell lines derived from elderly female patients (*p* = 0.04 from chi-squared test using 70 years as age threshold). Five MSS cell lines (5%; HCA24, HCC2998, HROC69, HT115 and SNU-81) had pathogenic *POLE* mutations and a higher mutational load (median of 18 non-synonymous mutations, range 18–27) than MSI (median of 9, range 4-15, *p* = 0.001) or *POLE* wild-type MSS cell lines (median of 4, range 0-9, *p* = 0.0002, both by Wilcoxon rank sum test; Fig. 1A) in 19 analyzed genes (excluding *POLE*). The hypermutation phenotypes (MSI and MSS *POLE*-mutated) were enriched with the transcriptomic intrinsic consensus molecular subtype (iCMS) 3 [38] (odds ratio 45.3, *p* = 1 × 10^-7^ from Fisher’s exact test; Supplementary Table 1). The variant repertoire of *POLE-*mutated cell lines consisted almost exclusively of SNVs (*n* = 100 of totally 101 variants, 99%; Supplementary Tables 4 and 5), with high proportions of C > A, C > T and T > G variants in TpCpT, TpCpG and TpTpT sequence contexts, respectively (Supplementary Fig. 1A), consistent with the COSMIC single base substitution signature 10 and a *POLE* hypermutator phenotype [39]. Conversely, MSI cell lines had higher proportions of indels (median of 30% and 17% of mutations per MSI and MSS cell line, respectively, *p* = 2 × 10^-3^ by Wilcoxon’s test), and the SNV spectrum included frequent C > T and T > C transitions consistent with the COSMIC signatures of defective DNA mismatch repair (Supplementary Fig. 1B). The large proportion of MSI-associated C > T transitions was accompanied by frequent C > A transversions in the context of CpCpT in HCT 15/DLD-1 and KM12, possibly attributed to concurrent MSI and *POLD1* mutations [40] (Supplementary Figs. 1 and 2, Supplementary Tables 6, 7 and 8). Notably, CL-40 has previously been reported as MSI [26] but was detected as MSS in our analyses [30], consistent with a low number of mutations (5 in 4 genes).

### Spectrum of common mutations

The mutation frequency of each of the 20 sequenced genes corresponded well with the patterns found across clinical cohorts of CRCs (Fig. 1A and Table 1) [38, 41–50]. The distribution of mutations along the length of the encoded protein is illustrated for each of 10 selected genes with high mutation frequency and according to the hypermutation status of the cell lines in Supplementary Fig. 3 (MSS *POLE* wild-type versus MSI and MSS *POLE*-mutated). The complete list of mutations and predictions of functional effects and pathogenicity according to Cell Model Passports, ClinVar, and OncoKB databases [51–53] is included in Supplementary Tables 9 and 10. Nearly all truncating mutations (97% of nonsense SNVs and frameshift indels) were found in tumor-suppressor genes (TSGs), while oncogenes were more frequently targeted by missense mutations (53%, *p* < 2.2 × 10^−16^ from Fisher’s test of truncating versus missense mutations in TSGs versus oncogenes). Non-hypermutated cell lines had a higher proportion of mutations with pathogenic effects (87%) than MSI (67%) or *POLE*-mutated cell lines (54%; *p* = 10 × 10^−12^), consistent with accumulation of passenger events in hypermutated cell lines (Fig. 1B). For example, 95% of non-synonymous mutations of *APC* in non-hypermutated cell lines were classified pathogenic, compared to only 64% and 63% in MSI and *POLE* mutated cell lines, respectively (*p* = 1 × 10^−6^, both by Fisher’s exact test). A notable exception was *TP53*, where nearly all mutations were classified as pathogenic in both groups of cell lines (96% and 100%, respectively).

**Table 1:** Mutation prevalence of each gene per mutation phenotype.

Gene	MSS cell lines (%), *n* = 76	MSI cell lines (%), *n* = 15	MSS-POLE mutated cell lines (%), *n* = 5
APC	64 (84)	13 (87)	5 (100)
ARID1A	8 (11)	11 (73)	3 (60)
BRAF	14 (18)	10 (67)	1 (20)
CCND2	0 (0)	1 (7)	1 (20)
CTNNB1	5 (7)	5 (33)	3 (60)
EGFR	3 (4)	2 (13)	2 (40)
ERBB2	1 (1)	3 (20)	1 (20)
FBXW7	15 (20)	5 (33)	5 (100)
JAK1	5 (7)	2 (13)	1 (20)
KRAS	43 (57)	6 (40)	2 (40)
MDM2	0 (0)	2 (13)	2 (40)
MYC	1 (1)	2 (13)	0 (0)
NF1	5 (7)	6 (40)	4 (80)
NRAS	3 (4)	0 (0)	2 (40)
PIK3CA	15 (20)	7 (47)	4 (80)
POLE	0 (0)	0 (0)	5 (100)
PTEN	2 (3)	4 (27)	4 (80)
SMAD2	4 (5)	3 (20)	3 (60)
SMAD4	19 (25)	2 (13)	1 (20)
TP53	64 (84)	6 (40)	4 (80)

Mutations are reported by excluding samples from the same patient and from neuroendocrine tumors.

A fairly large proportion of mutations (23%) involved multiple hits of the same gene in the same cell line. The distribution of such multi-hit mutations corresponded with the overall mutation frequency of the gene and/or the mutation phenotype of the sample. The majority were found in hypermutated cell lines (68%; *p* = 8.8 × 10^-9^ from Fisher’s test of MSI and *POLE*-mutated versus MSS *POLE* wild-type), and multi-hit mutations of genes other than *APC* or *TP53* were rare in non-hypermutated samples (*BRAF* p.V600E/p.T119S: HT29 and WiDr; *KRAS* p.Q61H/p.V14I: CL-11; *SMAD2* p.I414fs/p.R120*: Isreco-1). Multiple truncating mutations occurred in 54% of *APC*-mutated samples (*n* = 44), and these appeared to have a non-random distribution. In 93% of the affected samples, one of the truncating mutations occurred in the first 1262 amino acids of the *APC* gene product, upstream of all the 20-residues β-catenin-binding sites (APC_r, Fig. 1C, Supplementary Tables 11 and 12), and the second in the mutation cluster region (MCR, amino acids 1263-1587) [54], thus leaving at least one 20-residues β-catenin-binding site intact in one of the mutated *APC* alleles. This mutation pattern supports the “just-right” WNT/β-catenin signaling model proposed for colorectal tumorigenesis [55]. Across all genes and cell lines, the variant allelic frequency (VAF) of pathogenic multi-hit mutations was lower than that of single mutations (median 0.49, range 0.068–1 and median 1, range 0.076–1, respectively, *p* = 2.2 × 10^−16^). The VAF of single pathogenic mutations was higher in TSGs than in oncogenes (median 1, range 0.076 – 1 and median 0.56, range 0.1–1, respectively, *p* < 2.2 × 10^−16^ both by Wilcoxon’s test).

### Genetic interactions based on co-occurring and mutually exclusive mutations

**Fig. 2:**
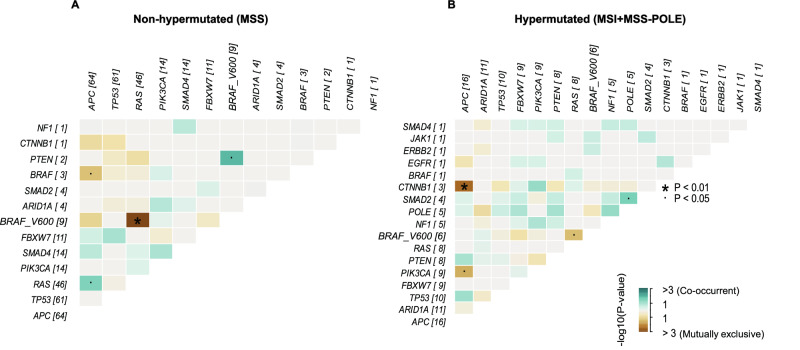
Somatic interactions of pathogenic mutations in gene pairs. Heatmap of significance levels for mutation interactions between gene pairs among **A** non-hypermutated (MSS; *n* = 76) and **B** hypermutated (MSI + MSS-POLE; *n* = 20) CRC cell lines. Co-occurrence versus mutually exclusivity is color-coded. *p*-values are from Fisher´s exact tests and plotted on log10-scale. Significant events are marked with asterisks (*p* < 0.01) and dots (*p* < 0.05) as indicated. Numbers next to the gene names correspond to the number of mutated samples. The *KRAS* and *NRAS* genes were grouped as *RAS*, and mutations targeting the *BRAF* V600 hotspot (p.V600E/K) were grouped separately from other *BRAF* mutations. Cell lines derived from non-unique patients (DLD-1, Isreco-3, SW62,0 and WiDr) and neuroendocrine cell lines (COLO 320, HROC57, NCI-H716) were excluded from the analysis. MSI/MSS: microsatellite instable/stable; MSS-POLE: *POLE* mutated samples.

Co-occurrence and mutual exclusivity of pathogenic mutations of gene pairs were analyzed in hypermutated (MSI and *POLE* mutant) and non-hypermutated samples separately (Fig. 2A, B). *BRAF* p.V600 mutations were mutually exclusive with *RAS* mutations in both sample groups. Pathogenic *BRAF* mutations generally had a negative interaction with *APC* mutations in non-hypermutated cell lines, but *BRAF* p.V600 co-occurred with truncating *APC* mutations in a subset (MDST8, COLO 205, HT-29/WiDr, and SW1417), modeling a particularly aggressive subtype of CRCs [56]. Pathogenic *APC* and *CTNNB1* mutations were mutually exclusive. *APC* mutations co-occurred with *RAS* in non-hypermutated cell lines and with *TP53* in hypermutated cell lines. Triple mutations of *APC*/*TP53*/*RAS* were found in 45% and 25% of non-hypermutated and hypermutated cell lines, respectively (*p* = 0.1 by Fisher’s exact test). Among the triple-mutated cell lines, 33% had two mutations in *APC* and one mutation in each of *TP53* and *RAS*, a combination that has been associated with poor prognosis in CRC [57]. Analyses across all cell lines suggested co-occurrence of pathogenic mutations of *PIK3CA* with *CTNNB1*, *NF1*, or *ARID1A*, as well as of *PTEN* with *SMAD2*, *NF1*, or *ARID1A* (Supplementary Fig. 4). These interactions were strongest among hypermutated cell lines, except the combination of *PIK3CA* and *ARID1A*.

### Copy number variations and allelic fractions of SNVs and indels

**Fig. 3:**
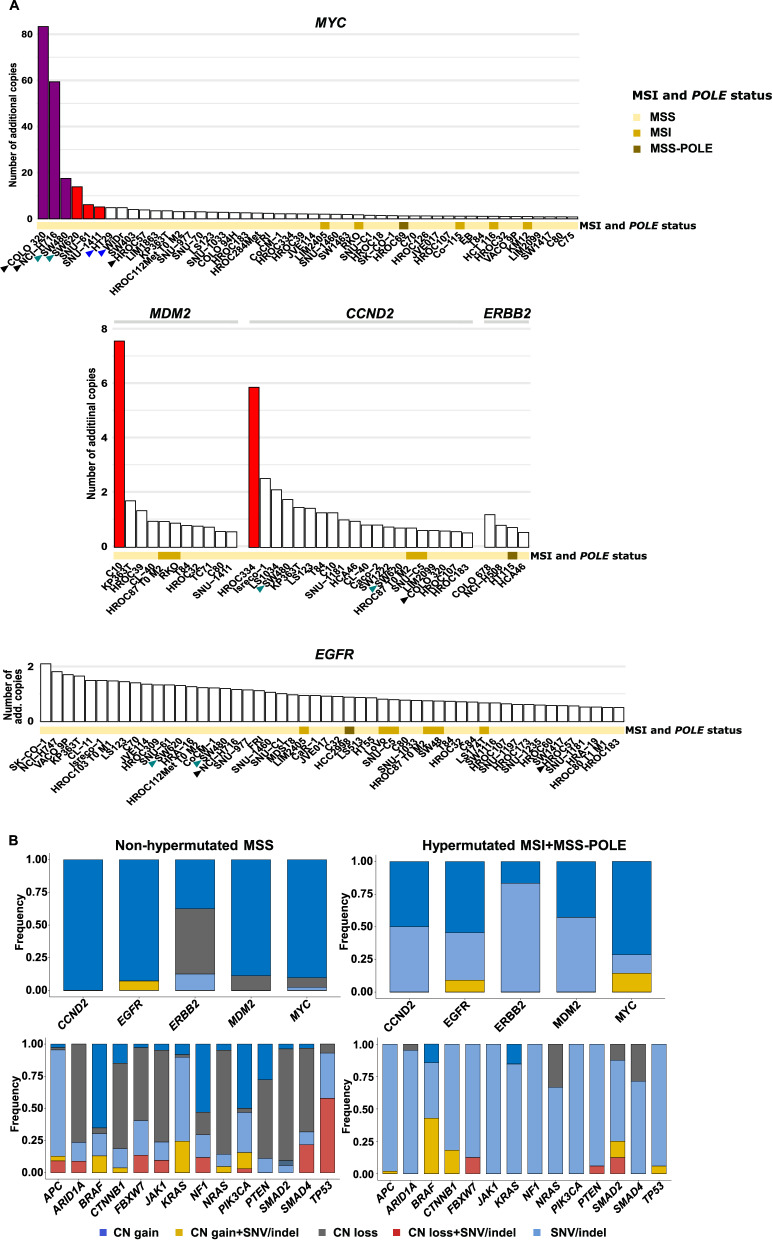
Gene amplifications and concurrent CNVs and SNVs/indels*.* **A** Barplot of amplifications and gains of *MYC*, *CCND2*, *MDM2*, *EGFR* and *ERBB2*. Low- and high-level amplifications are highlighted in red and magenta, respectively. Cell lines derived from the same patient (HT-29:WiDr, SW480:SW620) are marked by arrowheads of the same color. Cell lines derived from neuroendocrine tumors (COLO 320, HROC57, NCI-H716) are marked with black arrowheads. **B** Barplot of the proportion of different mutation types (CNVs and/or SNVs/indels) in each gene among non-hypermutated (MSS; left) and hypermutated (MSI + MSS-POLE; right) cell lines. MSI/MSS: microsatellite instable/stable; MSS-POLE: *POLE* mutated samples; CN gain: copy number gain; CN loss: copy number loss; SNV: single nucleotide variant; Indel: insertion or deletion.

*MYC* had high-level amplifications in COLO 320, NCI-H716, and SW480 (83, 5,9 and 18 copies, respectively) and low-level amplifications in SNU-1411, SNU-61, and SW620 (5, 6, and 14 copies, respectively; Fig. 3A top panel). *CCND2* and *MDM2* had low-level amplifications in one cell line each (6 and 8 copies in HROC334 and C10, respectively; Fig. 3A middle panel). Homozygous deletions of *SMAD4*, *PTEN*, *SMAD2* and *NF1* were found in 11 (14%), 3 (OUMS-23, SW1222, JVE127), 1 (COLO 94H) and 1 (HT55) MSS cell lines, respectively (Supplementary Fig. 5 and Supplementary Tables 13 and 14), although with the cautionary note that the gene panel was customized for CNV scoring only of the 5 genes shown in Fig. 3A. Nevertheless, our data confirm previous reports of homozygous deletions of parts of *SMAD4* in COLO 205 and SW403 [58–60] and complete homozygous deletions in COLO 678, HROC284Met, JVE017, KP363T, and SNU-1411. Our data also support complete homozygous deletion of *PTEN* in SW1222 and OUMS23 [27, 59, 61, 62], but suggest that the deletion in JVE127 involves only the first 5 exons of *PTEN* [61]. The latter is consistent with a fusion of *PAPSS2* and *PTEN*, involving breakpoints at the start of exon 5 of *PTEN* on one allele and exon 7 on the second [63].

The CNV frequency in relation to SNVs and indels of each gene is illustrated in Fig. 3B and Supplementary Fig. 6. *MYC*, *CCND2*, *MDM2*, and *EGFR* were primarily targeted by copy number gains, including in 45%, 19%, 11%, and 51% of the adenocarcinoma cell lines, respectively. *ERBB2* had gains and losses in the same proportions across cell lines (4%), and these did not occur with missense SNVs.

The CNV load per cell line was moderately inversely correlated with the SNV/indel load (Spearman´s rho - 0.41, *p* = 2.7 × 10^-5^), and the CNV load was higher in non-hypermutated cell lines (median 7, range 1–13) than in the hypermutated (median 2.5, range 1–5, *p* = 6.7 × 10^−8^ by Wilcoxon’s test). However, gains of genes on chromosome arms 7p, 7q, and 8q (*EGFR*, *BRAF*, and *MYC*, respectively) had similar frequencies between non-hypermutated (76%) and hypermutated cell lines (65%, *p* = 0.4 by Fisher’s exact test), consistent with previous studies [29, 30, 64, 65]. The higher CNV load was associated with higher VAFs of SNVs and indels in non-hypermutated (median 0.75, range 0.052-1) compared with hypermutated cell lines (median 0.48, range 0.051–1, *p* < 2.2 × 10^−16^ by Wilcoxon´s test; Supplementary Fig. 7A). Specifically, genes affected by copy number losses had higher VAFs of concurrent SNVs/indels (median 1 in non-hypermutated cell lines, range 0.42-1) than genes with gains or no CNVs (median 0.59, range 0.13-1 and median 0.64, range 0.05–1, respectively, *p* = 1.7 × 10^−14^, VAF for genes with gains and no CNVs relative to genes with losses, by Wilcoxon´s rank sum test). This reflected loss of heterozygosity at mutated loci and was primarily observed in TSGs (Supplementary Fig. 7B). However, SNVs/indels at copy number neutral loci also had higher VAFs in non-hypermutated versus hypermutated cell lines (median 0.64, range 0.05-1 and median 0.48, range 0.05–1, respectively, *p* < 2.2 × 10^−16^ by Wilcoxon´s rank sum test; Supplementary Fig. 8), indicating a stronger selection pressure on mutations not associated with hypermutation, as well as more frequent development of subclonal mutations with a low cellular prevalence in hypermutated cell lines. Homozygous mutations at copy number neutral loci (VAF > 0.99) were most frequently found in *APC* and *TP53*, including in 24% and 30% of the non-hypermutated cell lines, respectively.

## Discussion

This study reports the mutation profiles of 20 CRC-relevant genes in 103 conventional cell lines representing the three main genomic subtypes of CRC [3], including MSS-chromosomal instability, MSI, and MSS-*POLE* mutated. The cell lines recapitulated the mutation patterns of each phenotype, including the mutation load, relative proportion of mutation types (CNVs, SNVs, and indels), pathogenicity, allelic frequency, and co-mutations. Importantly, the STR profiles for each cell line are also made available in the study. The *POLE*-associated hypermutation phenotype was overrepresented in the cell line collection compared to primary tumors [22], and five cell lines with this rare subtype were included. Large pan-cancer cell line compendia, such as the cancer cell line encyclopedia [10] and the COSMIC cell line project [66, 67], describe genomic alterations in 56 and 55 CRC cell lines, respectively, with a 70% sample overlap with our collection. Accordingly, our study includes more than 60 additional CRC cell lines. Moreover, we extend the set of commonly analyzed genes in similarly-sized or larger CRC-only cell line datasets (beyond *NRAS*/*KRAS, BRAF, APC*, *PIK3CA*, *TP53*) [26, 27, 31, 32].

There was evidence of lower selection pressure and more frequent subclonality of mutations in cell lines with a hyper/ultramutation phenotype, observed as a lower proportion of pathogenic mutations and lower allelic frequencies. This highlights the need for particularly careful consideration of mutation relevance in studies involving this subgroup of cell lines. In contrast, most mutations in non-hypermutated cell lines were pathogenic and clonal. Homozygous or hemizygous mutations were largely restricted to this subgroup. This information should also be considered in functional studies, considering that the allelic frequency may impact the effects of mutations, including on drug sensitivity [68]. Notably, passage history impacts genetic drift, experimental reproducibility, and correspondence of cell line models to the tumor of origin [69], and the cell lines in our study were analyzed at low passage numbers. Loss of heterozygosity was a main cause of high allelic frequencies of SNVs/indels, and TSGs were more frequently affected than oncogenes. This was consistent with the more frequent targeting of TSGs also by loss-of-function (truncating) mutations, according to the “two-hit” hypothesis of recessive driver mutations [70]. Notably, low allelic frequencies of SNVs and indels at copy number neutral loci indicated subclonal evolution also in some of the non-hypermutated cell lines. As a resource to guide the selection of cell lines for functional studies, the allelic frequencies of all SNVs/indels and estimated DNA copy numbers of each gene are available in the supplementary material. The deep sequencing coverage is a strength of the study and should provide accurate estimates of allelic frequencies. A limitation of the study is the bias of the gene panel towards genes typically mutated in non-hypermutated CRCs, and well-known MSI target genes with short tandem repeats were not analyzed, such as *TGFBR2*. This might impact the relative distribution of mutations and mutation types among the different genomic phenotypes, including a smaller than expected difference in the total mutation load between MSI and MSS *POLE* wild-type cell lines. However, characteristics such as a higher frequency of indels in MSI and correspondence with the COSMIC base substitution signatures of both hypermutation phenotypes were also apparent with this gene panel. Although broader genomic coverage is needed to provide an unbiased representation of the mutation burden, the lack of matched nonmalignant reference samples to distinguish somatic mutations from germline variants is a challenge in genome-wide analyses of cell lines. Furthermore, CNV estimation was optimized for five genes known to be targeted by amplification in CRC, and the remaining copy numbers were estimated from exonic regions of each gene only. Nonetheless, the overall mutation profiles align with human tumors and previous cell line studies [26–30, 41–50], including reports of homozygous deletions of several TSGs [27, 58–62]. Co-occurring mutations further pinpointed representative models of clinically relevant subgroups of CRC. *APC* was frequently targeted by multiple truncating mutations in the same cell line, in accordance with the “just-right” WNT/β-catenin signaling model [55]. The number of mutations targeting *APC* is of clinical interest and has been suggested to have prognostic relevance in patients with CRC [57]. Furthermore, co-occurring mutations of *APC* with *KRAS* and *TP53*, or with *BRAF* p.V600, might identify poor-prognostic subsets of non-hypermutated CRCs [56, 57].

In conclusion, this study provides a detailed overview of the mutation profile of a 20-gene panel across a collection of 103 commonly used and authenticated CRC cell lines. All data are structured in tables and summary figures that allow the extraction of information for each cell line and gene, including estimates of allelic frequencies and significant co-mutations. This provides a resource to guide the selection of suitable models for functional studies in CRC.

## Material and Methods

### Cell lines

A total of 103 CRC cell lines were purchased from cell line repositories or kindly provided by collaborators (Supplementary Table 1). Cell lines were cultured in medium with added fetal bovine serum, antibiotics, and L-glutamine, and grown in a humidified 37 °C 5% CO_2_ incubator as previously described [28, 30]. All cell lines were routinely screened for mycoplasma infection using the MycoAlert Mycoplasma Detection Kit (Lonza, Basel, Switzerland) prior to collection. Genomic DNA was extracted using either standard phenol/chloroform extraction, a metallic beads protocol (Maxwell 16 DNA Purification Kit; Promega, Madison, WI, USA), or the AllPrep DNA/RNA/miRNA Universal Kit (Qiagen GmBH, Hilden, Germany). Determination of MSI status was done by analyzing the BAT-25/26 loci and/or by using the MSI Analysis System v1.2 (Promega, Madison, WI, USA). Cell line authenticity was confirmed by STR profiling using the AmpFLSTR Identifiler PCR Amplification Kit (Thermo Fisher Scientific, Waltham, MA, USA) and comparison to available STR profiles from the supplier. Percent STR match was estimated using the Cellosaurus STR Similarity Search Tool (CLASTR v1.4.4) [71].

### Gene panel design and targeted DNA sequencing

DNA sequencing of a custom panel of 20 genes (Supplementary Table 3) was performed using Twist Biosciences next-generation sequencing (NGS) Target Enrichment Solutions (San Francisco, CA, USA). Genes were selected based on mutation frequency and relevance in CRC, using information in the public repositories cBioPortal [72] and OncoKB [51], as well as in-house whole-exome sequencing data of primary CRCs [68, 73] and published data of metastatic CRCs [74]. Probes were designed to target all coding regions of all transcripts according to Gencode v32/ Ensembl Genes 101 (GRCh38) annotation, with the exception of *POLE*, for which probes were designed to target only known pathogenic exonuclease domain mutations (*n* = 11) [75]. The panel was optimized for CNV analyses of five genes by inclusion of probes targeting intronic regions. The distance between intronic probes varied according to intron length. The inter-probe distance was 1000 bp for *CCND2, ERBB2* and *MDM2*, 600 bp for *MYC*, 20,000 bp for the first intron of *EGFR*, and 2000 bp for all other introns of *EGFR*. The panel includes a total of 749 probes covering 478 target regions and 78,170 base pairs (bp). Notably, only target regions covered completely by probes have been analyzed in this study (*n* = 456 probes; 65,186 bp). Sequencing libraries were prepared from 50 ng DNA using the Twist Library Preparation Enzymatic Fragmentation (EF) kit and Twist Universal Adapter System, followed by target capture with custom probes and target purification using the Twist Binding and Purification Beads kit with Twist Universal Blockers as recommended in the Twist Target Enrichment Standard Hybridization protocol (Twist Biosciences). Enriched target libraries were submitted to 2 × 73 base-pair paired-end sequencing using the Illumina MiniSeq system and the Illumina MiniSeq High Output Reagent Kit (150-cycle; Illumina, San Diego, CA, USA).

### Sequence data processing and variant calling

Raw sequencing reads were quality controlled by the FastQC version 0.11.8 tool and aligned to the GRCh38 human reference genome with the Burrows-Wheeler Aligner (BWA) version 0.7.17 [76]. Aligned sequencing reads in Sequence Alignment Map (SAM) format were sorted, indexed, and converted to Binary Alignment Map (BAM) format using the SortSam function in Picard tools version 2.19.0 (http://broadinstitute.github.io/picard/). BAM files were refined with the Genome Analysis Toolkit (GATK) version 4.1.2 [77], including identification of duplicate reads using the MarkDuplicates function and base quality score recalibration using the BaseRecalibrator and ApplyBQSR functions. Single-nucleotide variants and indels were called with MuTect2 in tumor-only mode and annotated by ANNOVAR (version 2016-Feb-01) [78]. Non-synonymous exonic and splice site variants labeled as passed or clustered events were selected for further analyses and discarded if the total number of reads at the target locus was less than 15, the number of reads supporting the variant was less than 5, or the VAF was less than 5%. Candidate variants were filtered if listed in dbSNP (version avsnp144) but not in COSMIC version 76 or reported in the 1000 Genomes Project (v2014-oct) with VAF greater than 0.01. Variants with VAF greater than 0.45 and listed in dbSNP, reported in the 1000 Genomes Project, or annotated as clustered events were flagged for manual inspection.

Additional annotation of the mutations was retrieved from OncoKB using the Web application oncokb-annotator (https://github.com/oncokb/oncokb-annotator) and from the Ensembl database (hsapiens_gene_ensembl dataset version GRCh38.p14) using the Biomart package (v2.60.1) in R (v4.4.1). The latter included variant effect scores based on PolyPhen-2 [79] and SIFT [80]. Details on the scoring systems can be found at https://www.ensembl.org/info/genome/variation/prediction/protein_function.html. Classifications of variant pathogenicity were retrieved from Cell Model Passports [52], ClinVar [53], and OncoKB [51].

### Copy number analyses

CNVs were identified using MiniCN v0.1.0 (https://github.com/SveenLab/miniCN), an in-house R pipeline for DNA copy-number calling from small, amplicon-based targeted sequencing panels (<1 Mb). Coverage per sample and amplicon was estimated using DepthOfCoverage from GATK v3.6. To address the lack of matched normal samples, a pooled reference of 98 normal colonic mucosa samples from CRC patients was created based on mean amplicon coverage. MiniCN normalizes reads by dividing the read count per amplicon by the total read count of each sample. Copy number ratios are the normalized read counts per sample divided by the normalized read counts for the pooled reference. Correction for GC content was performed by fitting a local polynomial regression (LOESS) model to the log2 copy number ratios as a function of GC content in each amplicon using the “loess()” function, specifying family = “symmetric”, degree = 2, and surface = “direct” to compute an exact fitted surface. Gene-level CNVs were length-weighted averages of amplicon-level log2 copy number ratios, and statistical significance is assessed using Z-scores and Benjamini-Hochberg corrected q-values. High- and low-amplifications required at least two adjacent amplicons exceeding the respective thresholds (log2(17/2) and log2(7/2), equivalent to 15 and 5 additional copies). Deletions were runs of ≥ 2 adjacent amplicons with log2 ratios ≤ –2 (Supplementary Table 13). Gains and losses required statistical support (| Z | ≥ 3 and FDR *q*-value < 0.05) and ≥ 2 amplicons meeting the corresponding thresholds. All parameters were used as defaults, except that the thresholds for calling gains and losses were adjusted to log2(2.5/2) and log2(1.5/2), respectively.

### Statistics and plotting

Positive interactions (co-occurrence) and negative interactions of mutations in gene pairs were scored based on Fisher’s exact tests of mutation status and odds ratios above or below 1, respectively. Fisher’s exact tests, Wilcoxon’s tests, and Spearman’s correlation analyses were performed using RStudio v2024.4.1.748 (R v4.3.3), and *p*-values below 0.05 were considered significant. Statistical tests were two-sided. The choice of parametric or non-parametric tests was based on assessment of the normal distribution of the data using the Shapiro-Wilk’s test. Cell lines derived from non-unique patients (DLD-1, Isreco-3, SW620, and WiDr) and neuroendocrine cell lines (COLO 320, HROC57, NCI-H716) were excluded from analyses when indicated. Oncoplots, lollipop plots, and heatmaps were generated using the maftools R package (v2.18.0). The lollipop plot in Fig. 1C was generated with the g3viz (v1.2.0) package. Remaining plots were generated with ggplot2 (v3.5.1).

## Supplementary information

Supplementary Methods and Figures

Supplementary Tables

## Data Availability

All mutations (processed data) and STR profiles are available in the supplementary material.

## Code availability

The MiniCN R package, including source code, example data, and documentation, is freely available at https://github.com/SveenLab/miniCN. Additional computer code and supporting information used for data processing and plotting are accessible on Zenodo: 10.5281/zenodo.17357561.
