# Supplementary material for: Common gene mutations in 103 authenticated colorectal cancer cell lines
Source: Oncogenesis. 2026 Jan 27;15(1):8. doi: 10.1038/s41389-026-00599-0 (PMC12901148; doi:10.1038/s41389-026-00599-0)
Supplement: Supplementary file 1 — Supplementary Methods and Figures [file 41389_2026_599_MOESM1_ESM.pdf]

Supplementary Materials and Methods

Gene expression analysis

Transcriptomic profiling of all cell lines was performed with Affymetrix Human Transcriptome 2.0 arrays using 100 ng of total RNA as input and following the manufacturer's protocol (Thermo Fisher Scientific, Waltham, MA, USA). A subset of the samples has previously been published (*n* = 33) (1). Raw intensity CEL-files were processed according to the robust multi-array average approach (2) using the function justRMA in the R package affy (v. 1.84.0) (3) and custom CDF files from Brainarray (hta20hsentrezgcdf_25.0.0) (4). Gene annotations according to the GRCh38.p13 genome assembly were retrieved using the function getBM in the R package biomaRt (v. 2.58.0) (5). Protein-coding genes annotated with unique HGNC symbols were retained (*n* = 18,739). Classification according to the c onsensus molecular subtypes (CMS) was performed using the R package CMScaller (v. 2.0.1) with default settings (6). Classification according to the intrinsic CMS (iCMS) was performed using the approach and gene template described in the original publication (7).

Mutational signatures

Analyses of the COSMIC single base substitution signatures (8) were performed for MSI or POLE mutated cell lines based on somatic synonymous and non-synonymous substitutions in the gene panel data. Mutational signatures were detected using the web-based tool SigProfilerAssignment (9, 10). A VCF file with mutation data aligned to the GRCh38 human reference genome was used as input and matched to the COSMIC v3.4 reference set of mutational signatures (9).

Supplementary References

1. Berg KCG, Eide PW, Eilertsen IA, Johannessen B, Bruun J, Danielsen SA, et al. Multi-omics of 34 colorectal cancer cell lines - a resource for biomedical studies. Mol Cancer. 2017;16:116.

2. Irizarry RA, Hobbs B, Collin F, Beazer-Barclay YD, Antonellis KJ, Scherf U, et al. Exploration, normalization, and summaries of high density oligonucleotide array probe level data. Biostatistics. 2003;4:249–64.

3. Gautier L, Cope L, Bolstad BM, Irizarry RA. affy--analysis of Affymetrix GeneChip data at the probe level. Bioinformatics. 2004;20:307–15.

4. Dai M, Wang P, Boyd AD, Kostov G, Athey B, Jones EG, et al. Evolving gene/transcript definitions significantly alter the interpretation of GeneChip data. Nucleic Acids Res. 2005;33:e175.

5. Durinck S, Spellman PT, Birney E, Huber W. Mapping identifiers for the integration of genomic datasets with t he R/Bioconductor package biomaRt. N at Protoc. 2009;4:1184–91.

6. Eide PW, Bruun J, Lothe RA, Sveen A. CMScaller: an R package for consensus molecular subtyping of colorectal cancer pre-clinical models. Sci Rep. 2017;7:16618.

7. Joanito I, Wirapati P, Zhao N, Nawaz Z, Yeo G, Lee F, et al. Single-cell and bulk transcriptome sequencing identifies two epithelial tumor cell states and refines the consensus molecular classification of colorectal cancer. Nat Genet. 2022;54:963–75.

8. Sondka Z, Dhir NB, Carvalho-Silva D, Jupe S, Madhumita, McLaren K, et al. COSMIC: a curated database of somatic variants and clinical data for cancer. Nucleic Acids Res. 2024;52:D1210–D7.

9. Tate JG, Bamford S, Jubb HC, Sondka Z, Beare DM, Bindal N, et al. COSMIC: the Catalogue Of Somatic Mutations In Cancer. Nucleic Acids Res. 2019;47:D941–D7.

10. Diaz-Gay M, Vangara R, Barnes M, Wang X, Islam SMA, Vermes I, et al. Assigning mutational signatures to individual samples and individual somatic mutations with SigProfilerAssignment. Bioinformatics. 2023;39.

Supplementary Figures

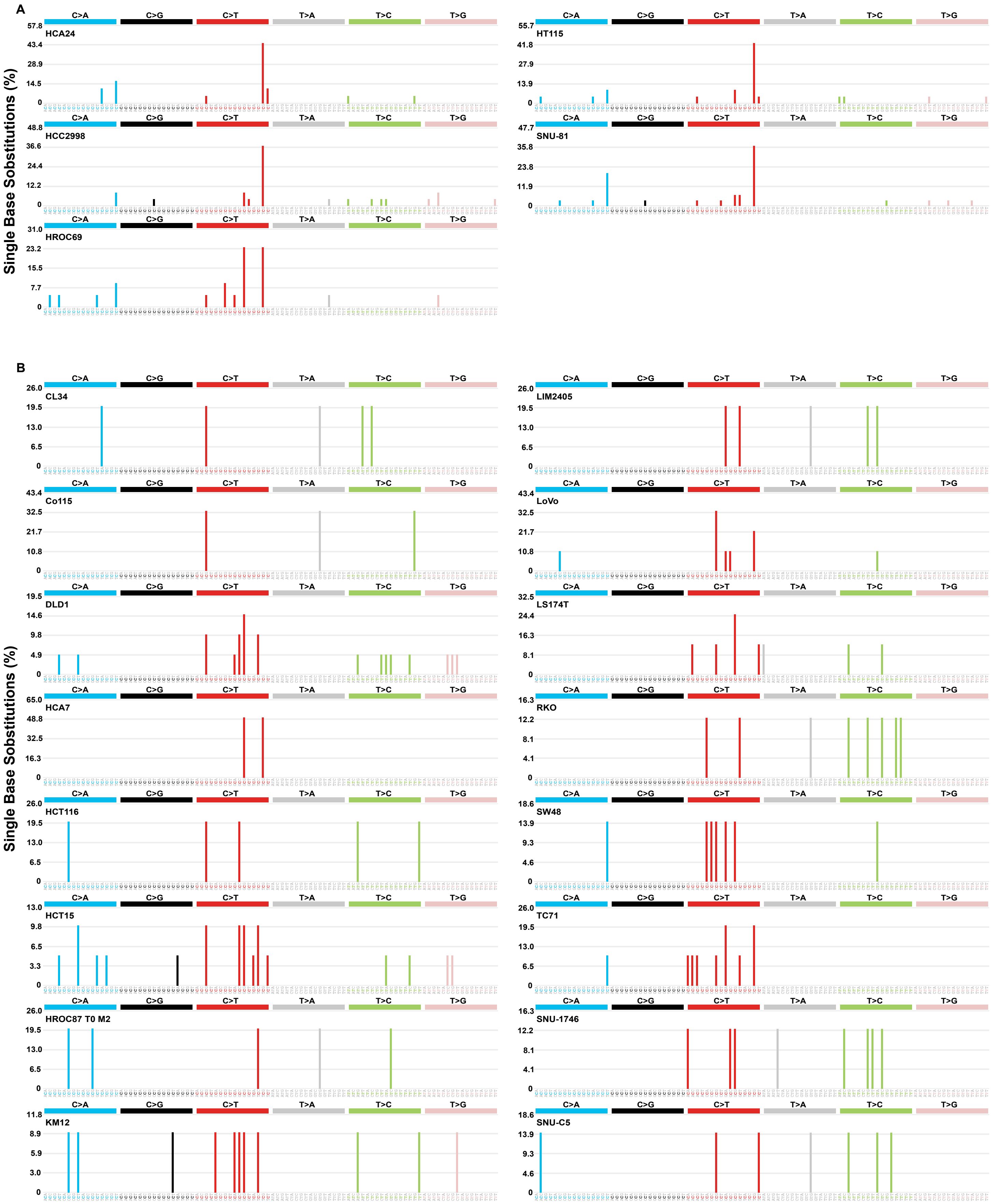

Supplementary Figure 1. Mutation signatures associated with hypermutation phenotypes. Frequencies (vertical axes) of base substitutions plotted for each cell line with pathogenic *POLE* mutations (*n* = 5) **A** or MSI (*n* = 16) **B** according to substitution type (indicated in the top panels and with color codes) and sequence context (flanking nucleotides are indicated on the horizontal axes).

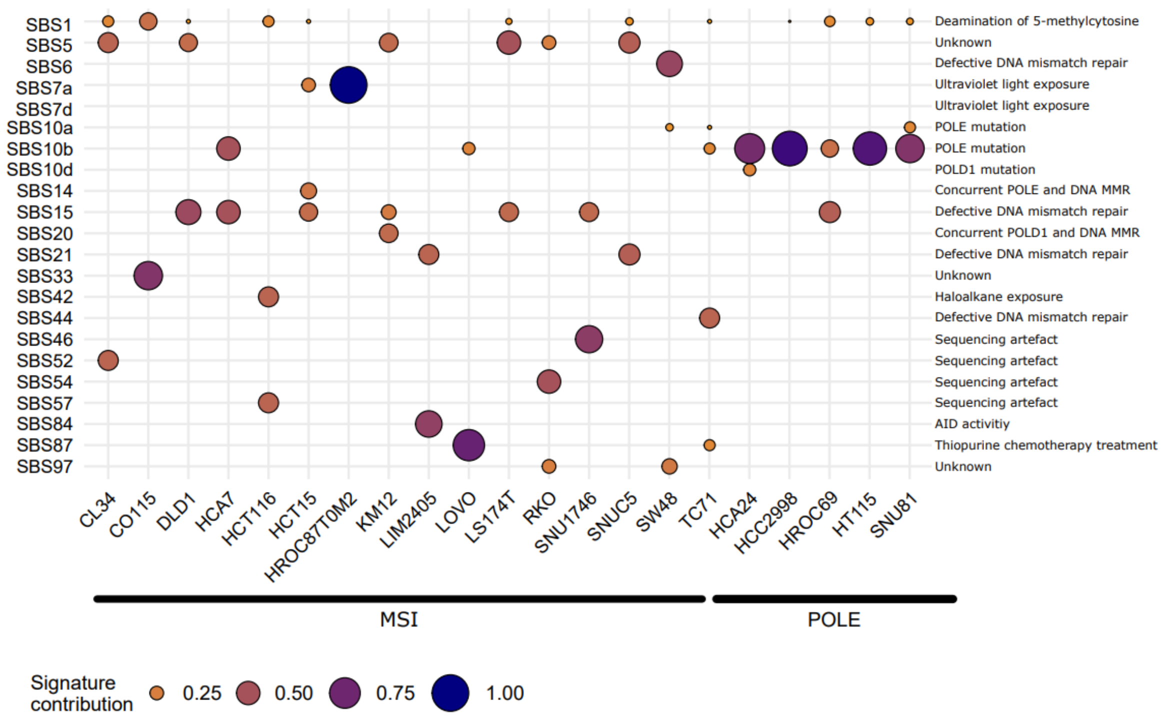

Supplementary Figure 2. Mutational signature contributions in hypermutated cell lines. Bubble plot showing the contribution of the listed single base substitution (SBS) signatures (left side) to the SNV load of each of the MSI and MSS *POLE* mutated cell lines. The proposed aetiology for each signature is shown on the right side.

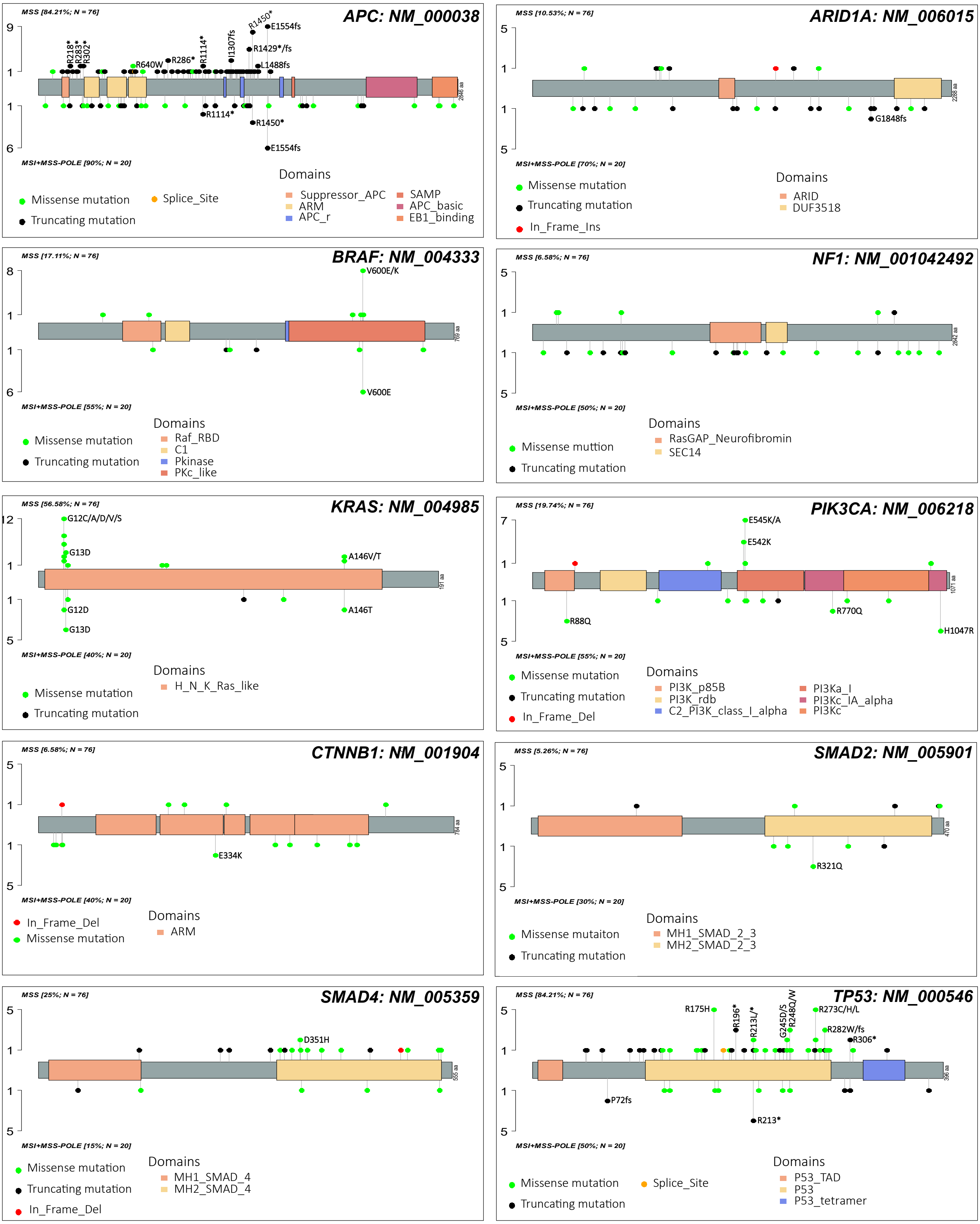

Supplementary Figure 3. Distribution of non-synonymous mutations along the encoded

protein sequence of frequently mutated genes. Lollipop plots of mutations in non-hypermutated cell lines (MSS; top) and hypermutated cell lines (MSI+MSS-POLE; bottom) for each of ten frequently mutated genes. Mutations are colored according to the mutation type, as indicated in each plot. Recurrent mutations occurring in 2 or more samples are specified. Protein domains are color-coded and described for each gene (Supplementary Table 10). Mutations in cell lines derived from non-unique patients (DLD-1, Isreco-3, WiDr, SW620) and in the neuroendocrine cell lines (COLO 320, HROC57, NCI-H716) are not included. MSI/MSS: microsatellite instable/stable; MSS-POLE: *POLE* mutated samples.

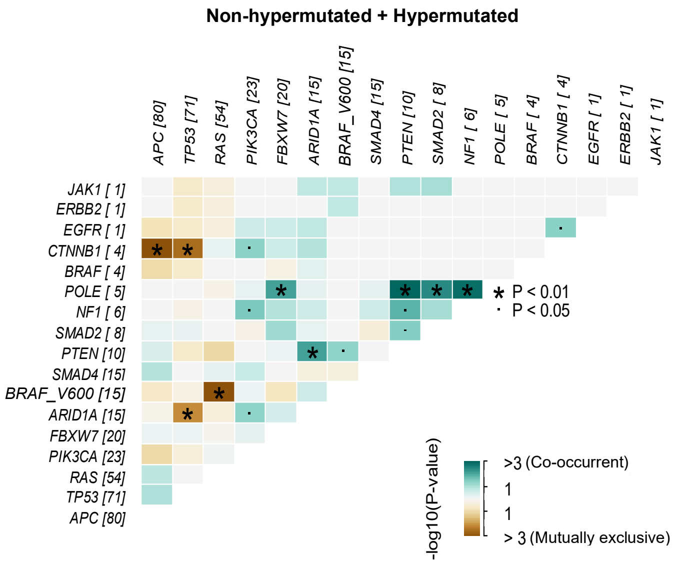

Supplementary Figure 4. Somatic interactions of pathogenic mutations across phenotypes. Heatmap of significance levels for mutation interactions between gene pairs in hypermutated and non-hypermutated cell lines (*n* = 96) together. Co-occurrence versus mutually exclusivity is color-coded. Statistical significance of co-occurrence and mutually exclusivity was determined as described in Figure 2. Significant events are marked with asterisks and dots as indicated. Numbers next to the gene names correspond to the number of mutated samples. The *KRAS* and *NRAS* genes were grouped as *RAS* and mutations targeting the *BRAF* V600 hotspot (p.600V>E/K) were grouped separately from other *BRAF* mutations. Cell lines derived from non-unique patients (DLD-1, Isreco-3, SW620 and WiDr) and neuroendocrine cell lines (COLO 320, HROC57, NCI-H716) were excluded from the analysis. MSI/MSS: microsatellite instable/stable; MSS-POLE: *POLE* mutated samples.

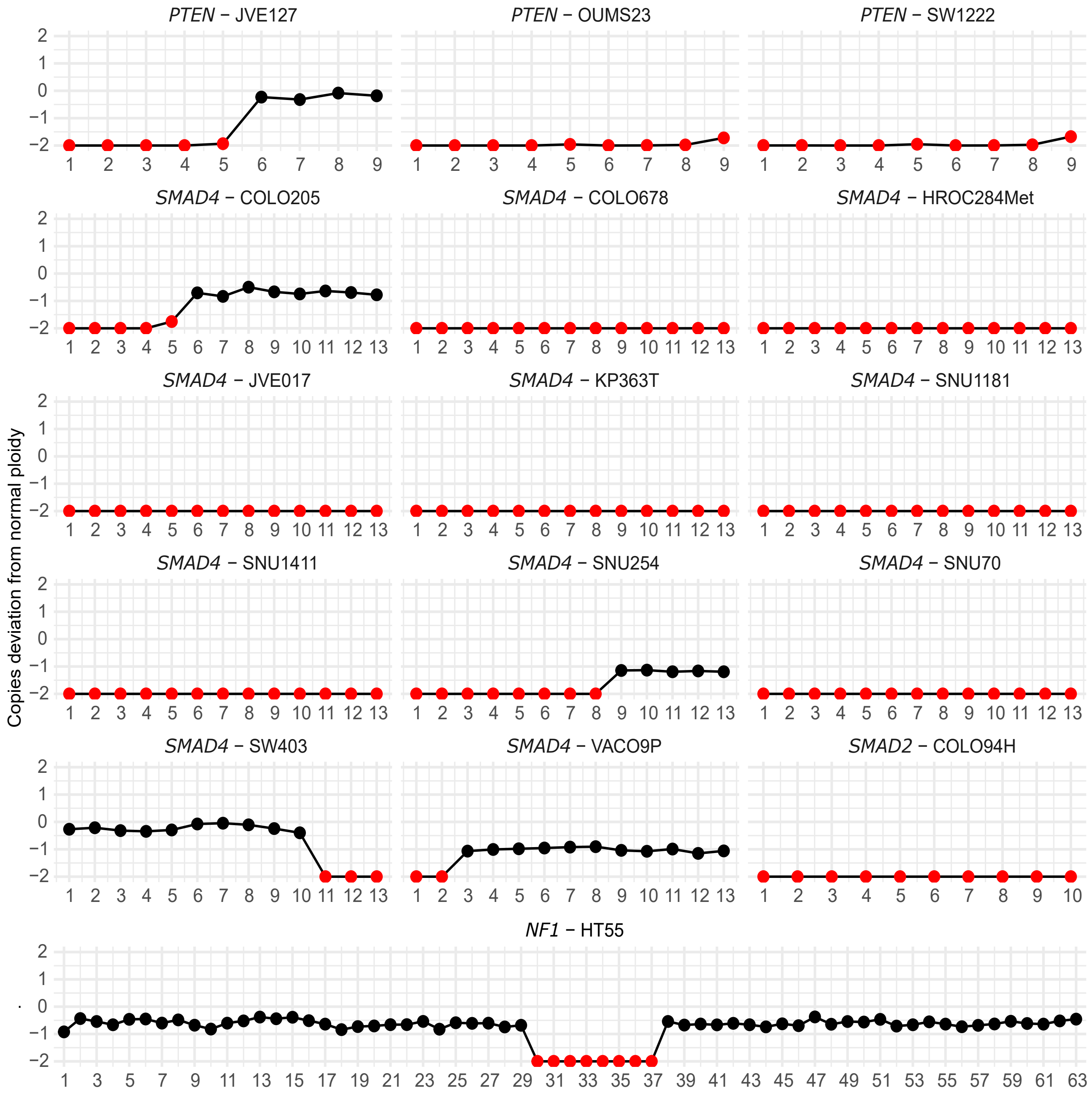

Supplementary Figure 5. Overview of homozygous gene deletions in the gene panel. The copy number deviation from the normal diploid genome (normal ploidy = 0 on y-axis) is displayed for *PTEN* and *SMAD2* (top four panels) and *SMAD4* (bottom set of panels) in cell lines for which partial or complete homozygous deletions have been detected (copy numbers range -1.54 to -2, in Supplementary Table 13). The values on the x-axis correspond to the amplicon index as indicated in Supplementary Table 14. The red segments mark the gene areas affected by the homozygous deletions.

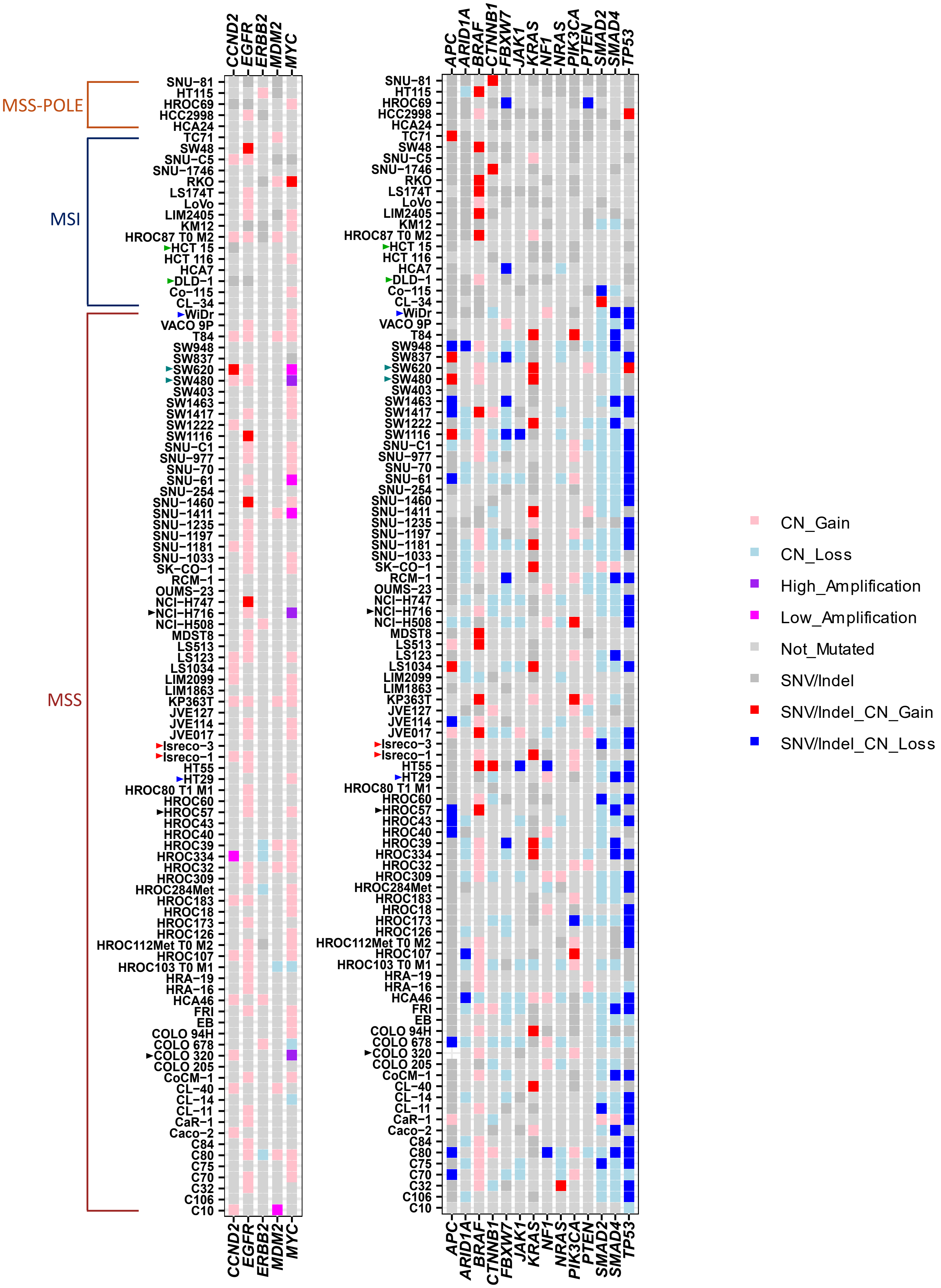

Supplementary Figure 6. Overview of concurrent DNA copy number alterations and

SNVs/indels in the gene panel. Copy number alterations detected in *MYC*, *CCND2*, *EGFR*, *MDM2* and *ERBB2* (left) and the rest of the gene panel (right) are color-coded according to the type of aberration and whether they co-occur with SNVs/indels in each sample. Cell lines are ordered alphabetically within each hypermutation phenotype. Cell lines derived from the same patient (DLD-1:HCT 15, Isreco-1:Isreco-3, HT-29:WiDr, SW480:SW620) are marked by arrowheads of the same color. Cell lines derived from neuroendocrine tumors (COLO 329, HROC57, NCI-H716) are marked with a black arrowhead. MSI/MSS: microsatellite instable/stable; MSS-POLE: *POLE* mutated samples; CN_Gain: copy number gain; CN_Loss: copy number loss; SNV: single nucleotide variant; Indel: insertion or deletion.

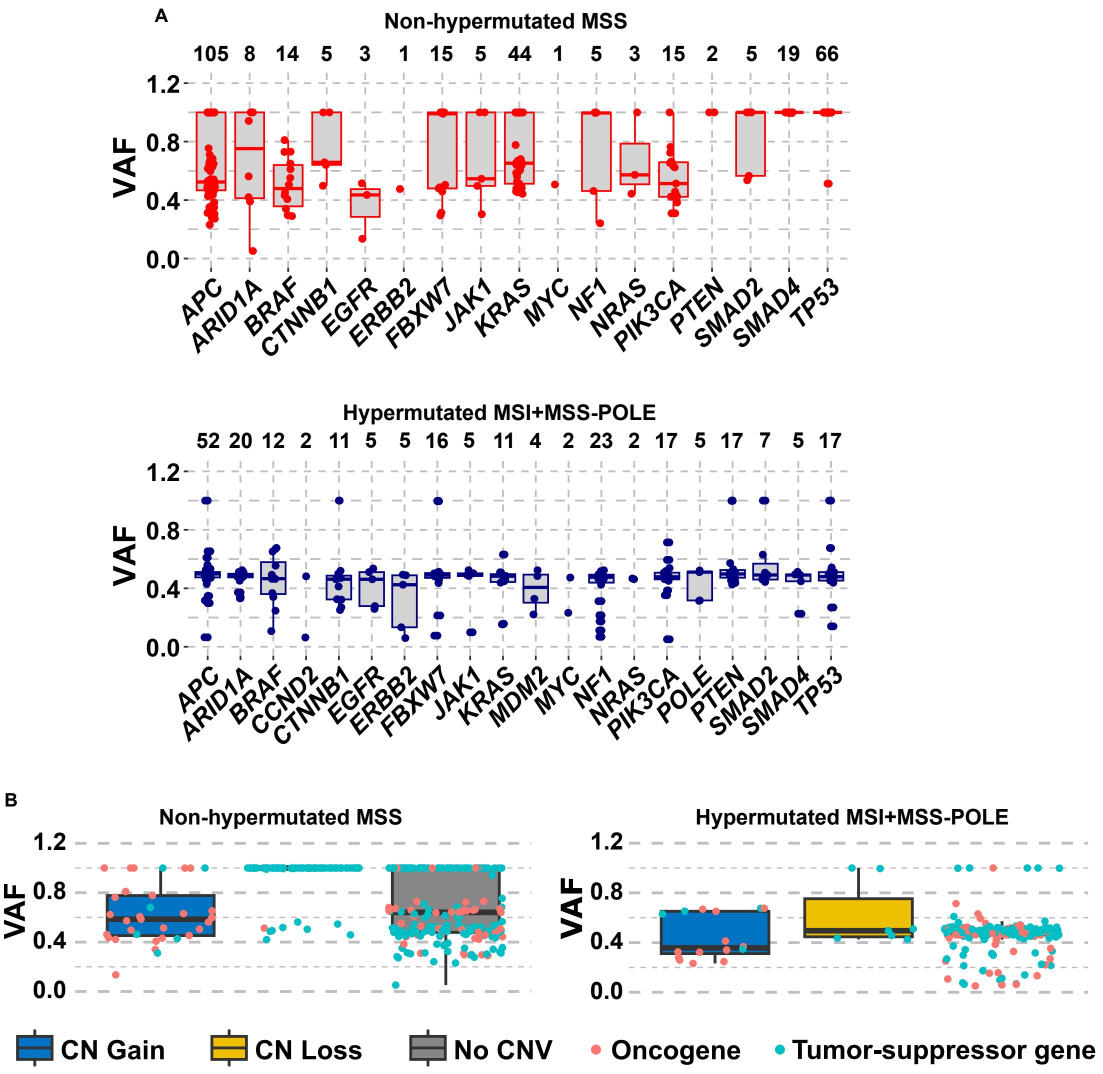

Supplementary Figure 7. Variant allelic frequencies across the gene panel. A Boxplots of the VAF of SNVs/indels in each gene plotted separately for non-hypermutated (MSS; top) and hypermutated (MSI+MSS-POLE; bottom) samples. Each dot in the plots represents a mutation and the number of mutations of each gene are indicated on the top. Mutations in cell lines derived from non-unique patients (DLD-1, Isreco-3, WiDr, SW620) and in the neuroendocrine cell lines (COLO 320, HROC57, NCI-H716) are not included. **B** Boxplot of the VAF of mutations according to the DNA copy number status of the mutated gene plotted separately for non-hypermutated (MSS; left) and hypermutated (MSI+MSS-POLE; right)

samples. Genes are classified as tumor-suppressors or oncogenes as described in Figure 1. Mutations in cell lines derived from the same patient (DLD-1, Isreco-3, WiDr, SW620) and in the neuroendocrine cell lines (COLO 320, HROC57, NCI-H716) are not included. MSI/MSS: microsatellite instable/stable; MSS-POLE: *POLE* mutated samples; CN gain: copy number gain; CN loss: copy number loss; No CNV : no copy number variation.

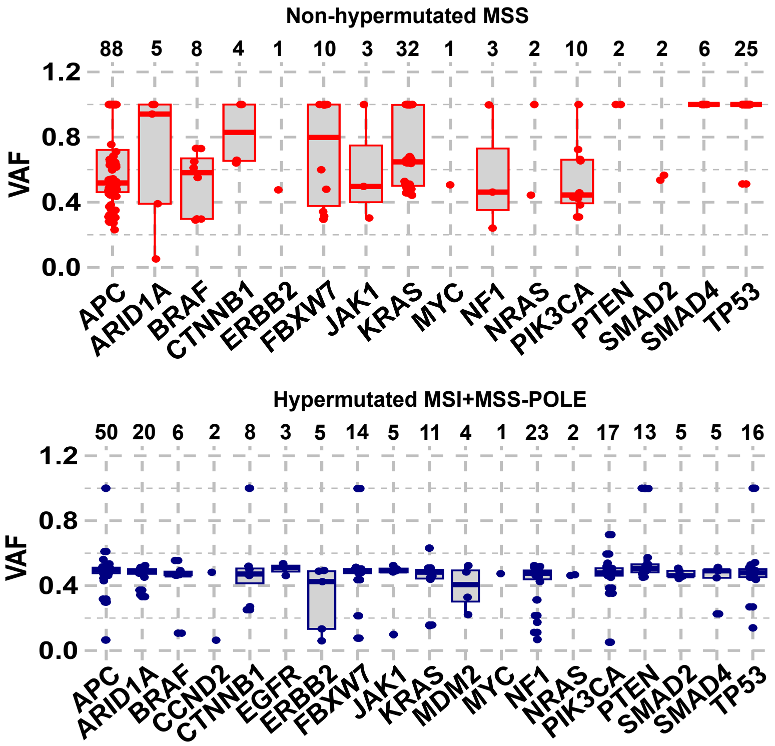

Supplementary Figure 8. Variant allelic frequencies in genes not affected by CNVs. Boxplots of VAFs for mutations not associated with CNVs in each gene were plotted separately for non-hypermutated (MSS; top) and hypermutated (MSI+MSS-POLE; bottom) samples. Each dot in the plots represents a mutation and the number of mutations for each gene are indicated on the top. Mutations in cell lines derived from non-unique patients (DLD-1, Isreco-3, WiDr, SW620) and in the neuroendocrine cell lines (COLO 320, HROC57, NCI-H716) are not included. MSI/MSS: microsatellite instable/stable; MSS-POLE: *POLE* mutated samples.
